# Bridging engineering and neuro-oncology: a scalable FastAPI-deployed CNN framework for real-time explainable brain tumor diagnosis

**DOI:** 10.3389/fnins.2026.1772429

**Published:** 2026-03-11

**Authors:** Sajjad Nematzadeh, Ferzat Anka, Fatih Ciftci, Kadriye Yasemin Usta Ayanoğlu, Ali Can Özarslan, Emir Oncu

**Affiliations:** 1 Department of Software Engineer, Engineering and Natural Sciences Faculty, Istanbul Topkapi University, Istanbul, Türkiye; 2 Data Science Application and Research Center (VEBIM), Fatih Sultan Mehmet Vakıf University, Istanbul, Türkiye; 3 Department of Biomedical Engineering, Faculty of Engineering, Fatih Sultan Mehmet Vakıf University, Istanbul, Türkiye; 4 Biomedical Electronic Design Application and Research Center (BETAM), Fatih Sultan Mehmet Vakıf University, Istanbul, Türkiye; 5 BioriginAI Research Group, Department of Biomedical Engineering, Fatih Sultan Mehmet Vakıf University, Istanbul, Türkiye; 6 Department of Tropiko Software and Consultancy, Istanbul, Türkiye; 7 Department of Distributed Software System, Faculty of Engineering, Technische Universität Darmstadt, Darmstadt, Germany; 8 Department of Metallurgical and Materials Engineering, Faculty of Engineering, Istanbul University-Cerrahpasa, Türkiye; 9 Department of Bioengineering, Faculty of Chemical and Metallurgical Engineering, Yıldız Technical University, Istanbul, Türkiye

**Keywords:** brain tumor, convolutional neural network, grad-CAM, machine learning, MRI classification

## Abstract

**Background:**

Automated and interpretable classification of brain tumors from MRI scans remains a critical challenge in medical imaging and neuro-oncology. This study addresses the need for reliable and deployable AI-driven tools that support timely tumor differentiation while maintaining transparency and practical usability.

**Methods:**

A deep learning–based diagnostic framework was developed using convolutional neural networks implemented in TensorFlow. The system was trained and evaluated on a curated dataset of 3,097 axial brain MRI images spanning four classes: glioma, meningioma, pituitary tumor, and normal cases. To ensure robust performance estimation, all models were evaluated using stratified 5-fold cross-validation and benchmarked against multiple state-of-the-art transfer learning architectures. For real-world applicability, the selected models were deployed via a FastAPI-based server, and Gradient-weighted Class Activation Mapping (Grad-CAM) was incorporated to provide qualitative visual explanations.

**Results:**

Across cross-validation folds, the proposed framework demonstrated stable and competitive performance in terms of accuracy, macro-averaged F1-score, and macro-averaged AUC, with low inter-fold variance. Comparative evaluation showed that transfer learning models achieved strong classification performance, while the lightweight custom CNN remained suitable for real-time deployment. The FastAPI implementation enabled low-latency inference and on-demand Grad-CAM visualizations, supporting transparent and responsive model usage.

**Conclusion:**

This work demonstrates the feasibility of bridging deep learning–based brain tumor classification with scalable, real-time deployment. By combining robust cross-validation, state-of-the-art benchmarking, and explainability-aware inference, the proposed framework provides a practical pathway toward integrating artificial intelligence into radiological workflows, while highlighting the importance of interpretability and deployment constraints in neuro-oncological applications.

## 1 Introduction

Brain tumors are abnormal growths of tissue within the cranial vault, where cellular proliferation becomes dysregulated and escapes normal physiological control mechanisms (Achrol et al., 2019). These tumors can originate primarily within the brain or represent secondary metastases from cancers elsewhere in the body. Clinically, brain tumors are classified into two major categories: benign (non-cancerous) and malignant (cancerous), with further subclassifications based on histological type, location, and grade (Ghorbian et al., 2024). Among many known types of brain tumors, gliomas, meningiomas, and pituitary adenomas represent some of the most frequently encountered, each exhibiting distinct biological behaviors and clinical challenges (Huttner, 2012).

Gliomas arise from glial cells and are broadly divided into low-grade and high-grade variants (Weller et al., 2024). Glioblastoma multiforme (GBM), the most aggressive form, is associated with rapid progression, significant infiltrative capacity, and a dismal prognosis despite multimodal therapy (Alifieris and Trafalis, 2015). Meningiomas, originating from arachnoid cap cells in the meninges, constitute the most common type of primary brain tumor (Bhat et al., 2014). Though often benign, their location and growth can lead to severe neurological deficits through compression of surrounding tissues. Pituitary tumors, generally benign adenomas located at the base of the brain, can severely disrupt endocrine function and exert pressure on nearby structures such as the optic chiasm, resulting in visual impairment and hormonal dysregulation (Carrie et al., 2018; Wang et al., 2024).

Accurate and early detection of brain tumors plays a pivotal role in improving patient outcomes, facilitating timely surgical intervention, radiotherapy, or chemotherapy (Naveen, 2024). However, due to the heterogeneous morphology, variable anatomical location, and overlapping radiological features of tumors, early diagnosis remains a formidable clinical challenge (Zhang et al., 2022). Magnetic Resonance Imaging (MRI) is the gold standard for non-invasive brain tumor assessment, offering superior contrast resolution and multiplanar imaging capabilities. Yet, the interpretation of MRI scans is time-consuming, requires expert radiologists, and may be prone to inter-observer variability (Montagne et al., 2021).

To overcome these limitations, Artificial Intelligence (AI) has emerged as a powerful tool in medical image analysis (Li et al., 2024; Gülmez, 2025). AI is rapidly emerging as an integral component of modern healthcare, with transformative applications in medical diagnostics that include the early detection and classification of brain tumors (Alam et al., 2024). In particular, Convolutional Neural Networks (CNNs) excel in learning complex spatial hierarchies from imaging data and have shown remarkable success in classifying, segmenting, and detecting pathological structures in various radiological modalities (Abdou, 2022; Zaman et al., 2025). Advancements in CNN architectures such as ResNet, VGG, Inception, and DenseNet have enhanced classification performance by capturing multi-scale features and reducing vanishing gradient problems (Jiao et al., 2025; Md Jelas et al., 2024; Younesi et al., 2024). Transfer learning further accelerates model development by adapting pre-trained networks to medical tasks, especially in data-constrained domains like MRI-based brain tumor detection (Chaki and Wozniak, 2023). Artificial Neural Networks (ANNs) have demonstrated significant potential in brain cancer detection by learning complex, non-linear patterns within medical imaging data, enabling accurate differentiation between healthy and tumorous brain tissues (Padmapriya et al., 2024; Islam et al., 2021). Additionally, hybrid approaches that integrate CNNs with recurrent neural networks (RNNs) or attention mechanisms are being explored to process sequential imaging data or highlight diagnostically relevant regions (Demiss and Elsaigh, 2024). Ben Brahim et al. (2024) proposed a new deep convolutional neural network (CNN)-based model for brain tumor detection using magnetic resonance imaging (MRI) data. The study aims to investigate the effectiveness of deep learning techniques in overcoming the challenges of early diagnosis of brain tumors. The proposed model ensures high accuracy in distinguishing tumorous tissues from healthy tissues by optimizing the image preprocessing and feature extraction stages. The experimental results obtained reveal that the developed model demonstrates superior performance compared to existing methods in the literature in terms of both classification accuracy and computational speed.

More recently, Transformer-based architectures and vision foundation models, such as Swin Transformers and large-scale medical image pretraining frameworks, have demonstrated state-of-the-art performance in various neuroimaging tasks, including brain tumor classification and segmentation. While these models offer strong representational power, they often introduce increased computational complexity, higher inference latency, and substantial deployment overhead, which may limit their applicability in real-time or resource-constrained clinical environments (Hatamizadeh et al., 2022; Chen et al., 2024; Zhou et al., 2019).

Considering these innovations, the integration of deep learning with neuroimaging offers a transformative approach to brain tumor diagnosis. A comprehensive systematic review analyzing over 1,100 studies has emphasized the pivotal role of AI and machine learning in brain tumor detection, particularly highlighting the outstanding diagnostic accuracy of CNN-based models while also underscoring critical challenges such as dataset bias, evaluation robustness, and the necessity of explainable AI for clinical adoption (Sajjad et al., 2019). Another study demonstrated the effectiveness of transfer learning by fine-tuning the YOLOv7 model for MRI-based brain tumor detection, achieving a remarkable 99.5% accuracy in identifying gliomas, meningiomas, and pituitary tumors, thereby underscoring the potential of deep learning in enhancing precision and localization in tumor diagnosis (Wang et al., 2023).

FastAPI is a modern, fast (high-performance) web framework for building APIs with Python 3.7+ based on standard Python type hints (Chen, 2023). In medical disease detection systems, FastAPI is commonly used to create efficient and scalable RESTful APIs that process and analyze medical data, such as images or sensor readings, enabling rapid and reliable disease diagnosis and prediction (Panch et al., 2018). While deep learning models such as MobileNet V2 and SSD have demonstrated remarkable accuracy in mobile applications for detecting COVID-19 pneumonia from chest X-ray images (Yellepeddi and Kuppusamy, 2024), there remains a notable gap between high-performing deep learning models and their systematic deployment in clinically usable, explainable, and scalable systems for neuro-oncology.

In this study, we propose a prediction system designed to classify brain MRI images into four categories: glioma tumor, meningioma tumor, pituitary tumor, and normal (non-tumorous) cases. Our model utilizes CNN-based architectures developed using TensorFlow and is evaluated through a robust cross-validation and benchmarking strategy against multiple state-of-the-art transfer learning models. Beyond model accuracy, the proposed framework emphasizes deployment feasibility, explainability, and reproducibility by integrating FastAPI-based real-time inference and Grad-CAM-based visual explanations. The system architecture supports rapid processing of MRI images uploaded by healthcare professionals, with predictions returned within seconds. Through this work, we aim to advance the practical applicability of CNN-based diagnostic tools for brain tumor classification, contributing toward the responsible integration of artificial intelligence into radiological workflows.

## 2 Materials and methods

For this study, a CNN model was developed to classify MRI images into four distinct categories, utilizing a dataset comprising 3,097 axial MRI images (Bhuvaji et al., 2020). To ensure robust and unbiased performance evaluation, the dataset was not evaluated using a single fixed split. Instead, a stratified 5-fold cross-validation protocol was employed, as detailed in “Section 2.2.”

The model was implemented using Python 3.12 and the TensorFlow deep learning library, chosen for its flexibility and efficiency in handling image-based data. The CNN model architecture was optimized to interpret the features in MRI images that are crucial for identifying and differentiating brain tumors. The primary goal of this model was to classify MRI images into the correct categories, thereby providing a rapid, automated method for assisting radiologists and other medical professionals in diagnostic decision-making. The following sections will detail the structure of the dataset, including class distribution and distinguishing features, and elaborate on the processing techniques and training methodologies applied.

### 2.1 Datasets and FastAPI-based system

The dataset utilized in this study consists of T1 and T2 weighing 3,097 MRI images divided into four classes: glioma tumor, meningioma tumor, normal, and pituitary tumor. Each class represents a distinct type of brain condition, which the CNN model aims to classify accurately. The dataset’s diversity enhances the model’s ability to generalize across different variations of brain conditions visible in MRI scans. These MRI scans were acquired using standard clinical imaging protocols, capturing axial views of the brain to ensure optimal visibility of tumor structures. As patient-level identifiers are not provided with the dataset, each MRI slice is treated as an independent sample. This unit of randomization is explicitly acknowledged and discussed as a limitation in the Discussion section. The images reflect a wide range of tumor sizes, anatomical locations, and morphological characteristics, representing realistic diagnostic scenarios. All images were resized to a fixed resolution of 224 × 224 pixels to ensure architectural compatibility across different CNN and transfer learning models. Data preprocessing steps included resizing and model-specific normalization, ensuring consistency across training and evaluation. This organized dataset enabled the CNN model to learn intricate details, leading to higher accuracy in classification tasks.

The FastAPI-based system developed in this study enables real-time classification of brain MRI images through an accessible web interface. Upon uploading an image, the system processes it using a pre-trained deep learning model to predict the class of the brain scan along with a corresponding confidence score. The backend architecture is constructed using FastAPI, a high-performance Python web framework, which efficiently handles HTTP requests, manages file inputs, and returns structured JSON responses. The prediction workflow involves loading the trained model, preprocessing the incoming image (resizing, normalization, etc.), performing inference, and returning the result to the user. This deployment-oriented design supports low-latency inference and scalable integration into clinical or research environments.

### 2.2 Machine learning model

A convolutional neural network was developed to perform multi-class classification of MRI images, producing both class predictions and qualitative explainability outputs via Grad-CAM. All models were trained and evaluated under an identical stratified 5-fold cross-validation protocol to ensure fair comparison and robust performance estimation.

**Figure 1:**
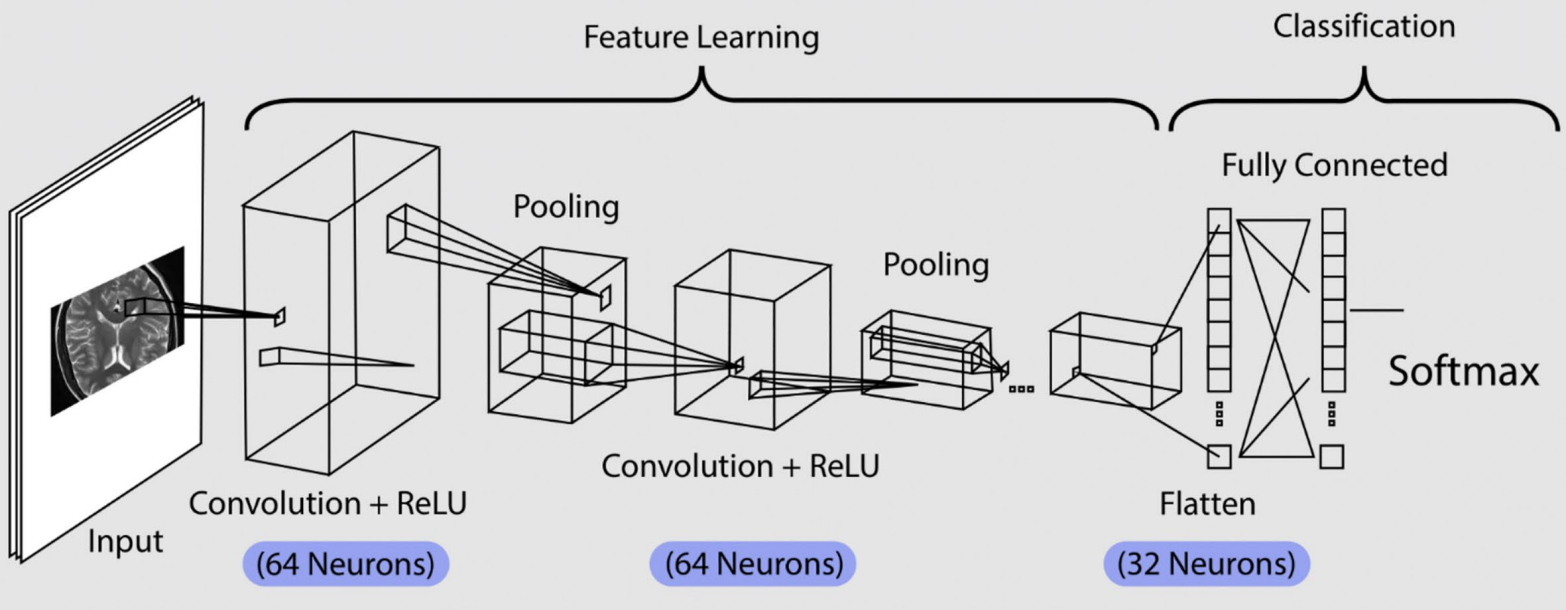
Convolutional neural network architecture for image classification.

For the custom CNN baseline, input images were standardized to 224 × 224 pixels, and a batch size of 16 was used to balance computational efficiency with convergence stability under CPU-only constraints. Transfer learning models followed the same input resolution and evaluation protocol to maintain methodological consistency (see Figure 1).

The model architecture uses TensorFlow’s Sequential API to build a multi-layer CNN. To preprocess the images, a Rescaling layer normalizes pixel values between 0 and 1, enhancing model stability and speeding up convergence. Additionally, a data augmentation layer randomly flips and rotates the images, a technique that increases the model’s generalization by reducing overfitting to specific features in the training data.

The model consists of multiple convolutional layers, starting with a layer of 64 filters and then expanding to 128 filters in subsequent layers. Each convolutional layer uses a 3×3 kernel size and ReLU activation function, which effectively captures spatial features while maintaining computational efficiency. Max-pooling layers are interspersed between these convolutional layers, down-sampling the feature maps and reducing dimensionality to focus on key spatial features. This layered structure allows the model to learn hierarchical features, beginning with low-level edges and textures and progressing to complex patterns relevant to classification. Once the features are extracted, a Flatten layer converts them into a 1D vector, which is then passed to fully connected (dense) layers for final classification. The first dense layer has 64 neurons and uses ReLU activation, capturing high-level representations from the convolutional layers. The output layer has a SoftMax activation function, producing probabilities for each of the four classes in the classification task.

To enhance interpretability, Gradient-weighted Class Activation Mapping (Grad-CAM) was applied to generate heatmaps highlighting image regions that most strongly influenced model predictions. The Grad-CAM analysis is qualitative and exploratory in nature and is intended to provide visual insight into the model’s decision-making process rather than clinical validation.

#### 2.2.1 Architecture selection

To justify the Custom CNN architecture, three configurations were evaluated:

- CustomCNN-Small: Reduced filter depth to assess underfitting risk and parameter efficiency.
- CustomCNN (Baseline): Balanced configuration with moderate depth and progressive feature expansion.
- CustomCNN-Deeper: Additional convolutional capacity to assess potential performance gains from increased representational depth.

All variants were evaluated under identical 5-fold stratified cross-validation settings. The Small configuration demonstrated reduced macro-F1 performance, suggesting insufficient feature extraction capacity. Conversely, the Deeper variant did not provide consistent performance improvement despite increased architectural complexity. The Baseline configuration achieved the highest macro-F1 and macro-AUC while maintaining moderate parameter count, representing the most favorable performance–efficiency trade-off for deployment-oriented applications (Table 1).

**Table 1:** Ablation study results for Custom CNN variants (5-fold stratified CV).

Model	Accuracy (mean ± std)	Macro-F1 (mean ± std)	Macro-AUC (mean ± std)	Parameters
Custom CNN-Small	0.728 ± 0.028	0.720 ± 0.039	0.917 ± 0.015	6,516,100
Custom CNN (Baseline)	**0.778 ± 0.034**	**0.776 ± 0.036**	**0.937 ± 0.017**	**5,761,348**
Custom CNN-Deeper	0.748 ± 0.030	0.750 ± 0.029	0.925 ± 0.016	3,729,988

## 3 Results

The architecture depicted in the image represents a CNN designed for image classification, structured into two main stages: feature learning and classification. Starting with the input layer, this model processes an image, such as an MRI scan, which is then passed through various layers to extract relevant information for classification. This input is essential as it initiates the flow of data, enabling the network to analyze pixel values and recognize patterns within the image. We modeled and implemented a sequential CNN consisting of four primary layers in Python using TensorFlow and Keras libraries. In addition to the custom CNN, multiple state-of-the-art transfer learning architectures were evaluated under identical experimental conditions to enable fair benchmarking. After training the CNN models, their learning behavior was examined through accuracy and loss curves. These visualizations provide insight into how effectively the models learned from the training data and generalized to unseen validation samples over successive epochs.

**Figure 2:**
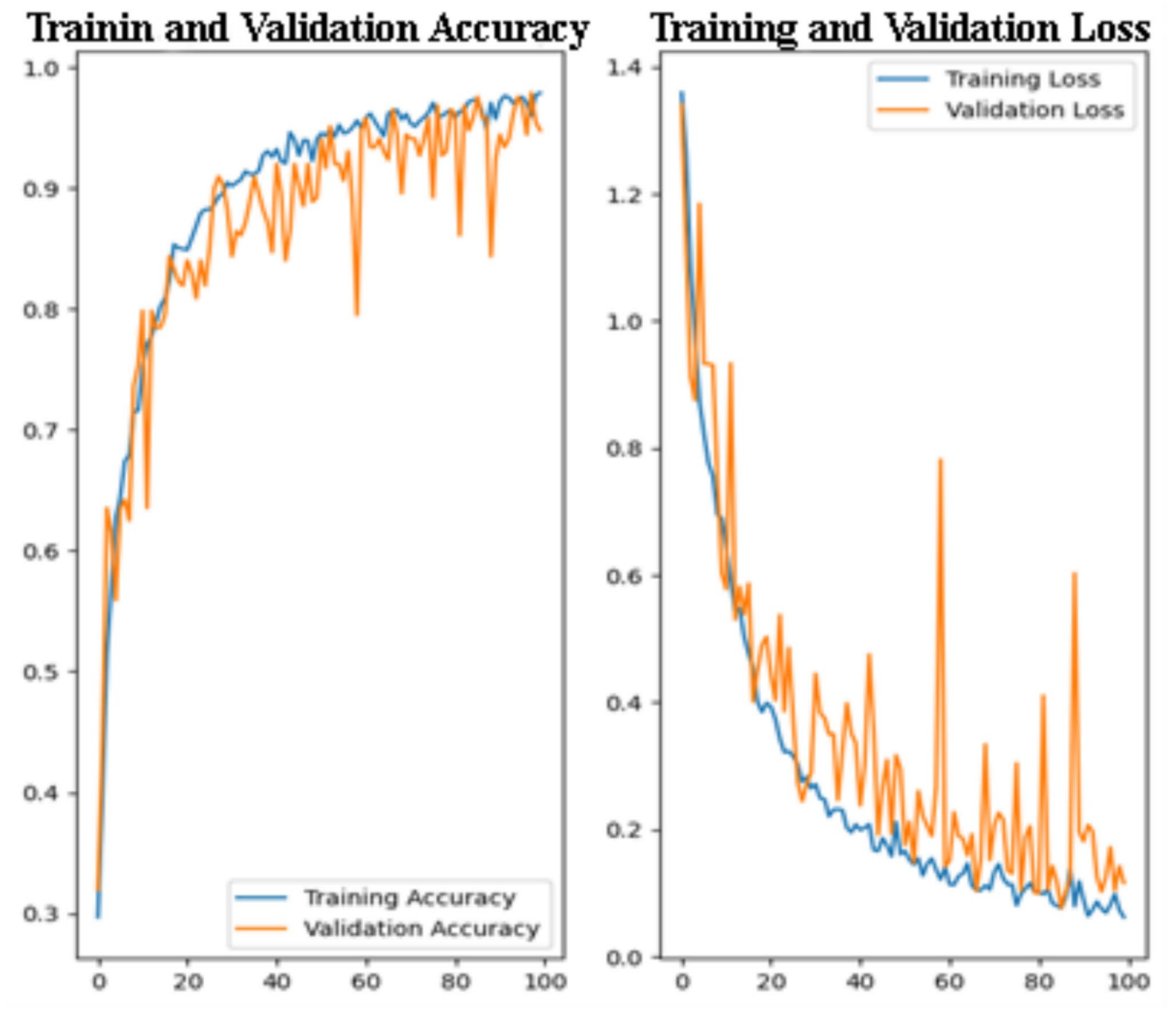
Training and validation accuracy and loss curves for CNN model.

Figure 2 displays the training and validation accuracy (left) and loss (right) curves over 100 epochs, providing insight into the learning behavior and generalization performance of the model. Although the models were trained with a maximum epoch limit, convergence was consistently achieved earlier due to early stopping, indicating stable optimization. On the left, the training and validation accuracy curves show a consistent upward trend, indicating that the model gradually learns and improves its performance on both the training and unseen validation data. The training accuracy starts at around 30% and steadily increases, surpassing 95% by the end of the training process. Similarly, the validation accuracy exhibits a sharp rise during the early epochs and stabilizes above 90% in the later epochs. Although the validation accuracy shows minor fluctuations, it remains consistently close to the training accuracy, suggesting minimal overfitting and strong generalization capability.

On the right, the training and validation loss curves exhibit a steady decrease throughout the training process. The training loss decreases smoothly, reflecting the model’s effective optimization. The validation loss also shows a general downward trend, although with more noticeable fluctuations. The close alignment between training and validation curves suggests limited overfitting and stable generalization across folds. Similarly, both training and validation loss curves exhibit a decreasing trend, reflecting effective feature learning. Minor fluctuations in validation loss are expected in medical imaging tasks due to inter-class similarity and imaging variability.

After analyzing the learning behavior of the baseline CNN, we further conducted an ablation study to justify the architectural design choices. Three configurations were evaluated under identical 5-fold stratified cross-validation settings: CustomCNN-Small (reduced capacity), CustomCNN (baseline), and CustomCNN-Deeper (increased depth). The comparative performance results are summarized in Table 1. The Small configuration was designed to assess the risk of underfitting by reducing representational capacity. The Deeper configuration was introduced to evaluate whether increasing architectural depth would improve discriminative performance. All training conditions, including optimizer settings, learning rate, batch size, and early stopping criteria, were kept constant to ensure a fair comparison.

As summarized in Table 1, the baseline CustomCNN achieved the highest macro-F1 and macro-AUC while maintaining moderate parameter complexity. The reduced-capacity model exhibited lower performance, indicating insufficient feature extraction capacity for reliable tumor discrimination. Conversely, the deeper configuration did not yield consistent improvements despite increased architectural complexity, suggesting diminishing returns beyond the selected depth. These findings demonstrate that the baseline configuration represents the most favorable balance between predictive performance and computational efficiency, supporting its selection for deployment-oriented evaluation.

### 3.1 Cross-validation performance and SOTA comparison

To ensure statistically robust evaluation, all quantitative results were obtained using stratified 5-fold cross-validation. Performance metrics are reported as mean ± standard deviation, along with 95% confidence intervals. This approach mitigates optimistic bias associated with single data splits and provides a more reliable estimate of real-world performance.

Table 2 summarizes the cross-validation results of the proposed custom CNN and four state-of-the-art convolutional neural network architectures evaluated using transfer learning. Performance is reported in terms of mean accuracy, macro-averaged F1-score, and macro-averaged AUC across the five folds, along with standard deviation values that reflect inter-fold variability. The results indicate that transfer learning–based architectures consistently outperform the custom CNN baseline, benefiting from pretrained feature representations learned from large-scale natural image datasets. Among the evaluated models, ResNet50 achieves the highest overall performance, demonstrating the strongest balance between feature expressiveness and generalization capability. DenseNet121, MobileNetV2, and EfficientNetB0 follow closely, exhibiting comparable macro-F1 and macro-AUC scores with minor variations across folds.

**Table 2:** 5-fold cross-validation performance comparison (mean ± std., 95% CI).

Model	Accuracy (mean ± std)	Macro-F1 (mean ± std)	Macro-AUC (mean ± std)
ResNet50	0.835 ± 0.017	0.843 ± 0.015	0.965 ± 0.006
DenseNet121	0.809 ± 0.015	0.817 ± 0.017	0.953 ± 0.009
MobileNetV2	0.808 ± 0.018	0.817 ± 0.019	0.955 ± 0.009
EfficientNetB0	0.800 ± 0.023	0.807 ± 0.019	0.956 ± 0.008
Custom CNN	0.739 ± 0.039	0.744 ± 0.042	0.919 ± 0.018

Despite its simpler architecture and substantially lower parameter count, the custom CNN achieves competitive performance, particularly in terms of macro-AUC. This suggests that the model is able to capture discriminative tumor-related features even when trained from scratch on a relatively limited dataset. However, the higher standard deviation observed for the custom CNN reflects increased sensitivity to data partitioning, which is expected for models without pretrained initialization. Importantly, all evaluated models demonstrate low inter-fold variance, indicating stable performance across different data splits. This consistency supports the robustness of the cross-validation protocol and confirms that the reported metrics are not driven by a particular fold or favorable data configuration.

Overall, these results highlight a clear trade-off between classification performance and deployment complexity. While deeper transfer learning models achieve superior accuracy and F1-scores, the lightweight custom CNN offers a favorable balance between performance, computational efficiency, and suitability for real-time deployment scenarios. This trade-off is particularly relevant in resource-constrained clinical environments, where inference latency and system scalability are critical considerations. Beyond aggregated metrics, a detailed class-wise evaluation was performed to examine tumor-specific discrimination behavior across folds. Table 3 presents fold-averaged precision, recall, and F1-scores for each class, providing clinically meaningful insight beyond global accuracy measures.

**Table 3:** Fold-averaged class-wise performance of the Custom CNN (5-fold stratified CV, mean ± std).

Class	Precision (mean ± std)	Recall (mean ± std)	F1-score (mean ± std)
Glioma	0.844 ± 0.073	0.734 ± 0.086	0.781 ± 0.053
Meningioma	0.685 ± 0.042	0.687 ± 0.074	0.686 ± 0.047
Normal	0.803 ± 0.079	0.742 ± 0.090	0.769 ± 0.069
Pituitary	0.834 ± 0.052	0.923 ± 0.041	0.875 ± 0.024

Values averaged across five stratified cross-validation folds.

The results indicate stable classification performance across all classes. Pituitary tumors achieved the highest recall, demonstrating strong sensitivity for this category. Glioma and normal classes exhibited balanced precision–recall trade-offs, indicating consistent discrimination capability. Slightly lower performance was observed for the meningioma class, which may reflect known radiological similarities between meningioma and glioma tumors. Importantly, no class exhibited systematic performance degradation across folds, confirming that the model’s predictive behavior is not driven by dominance of a single category but reflects consistent multi-class discrimination capability. These class-wise findings complement the aggregated cross-validation analysis and provide clinically meaningful insight into tumor-specific performance patterns.

Collectively, the results highlight a clear trade-off between classification performance and deployment complexity. While deeper transfer learning models achieve superior accuracy and macro-F1 scores, the lightweight custom CNN offers a favorable balance between predictive performance, computational efficiency, and suitability for real-time deployment scenarios. This trade-off is particularly relevant in resource-constrained clinical environments, where inference latency and system scalability are critical considerations.

### 3.2 Confusion matrix analysis

**Figure 3:**
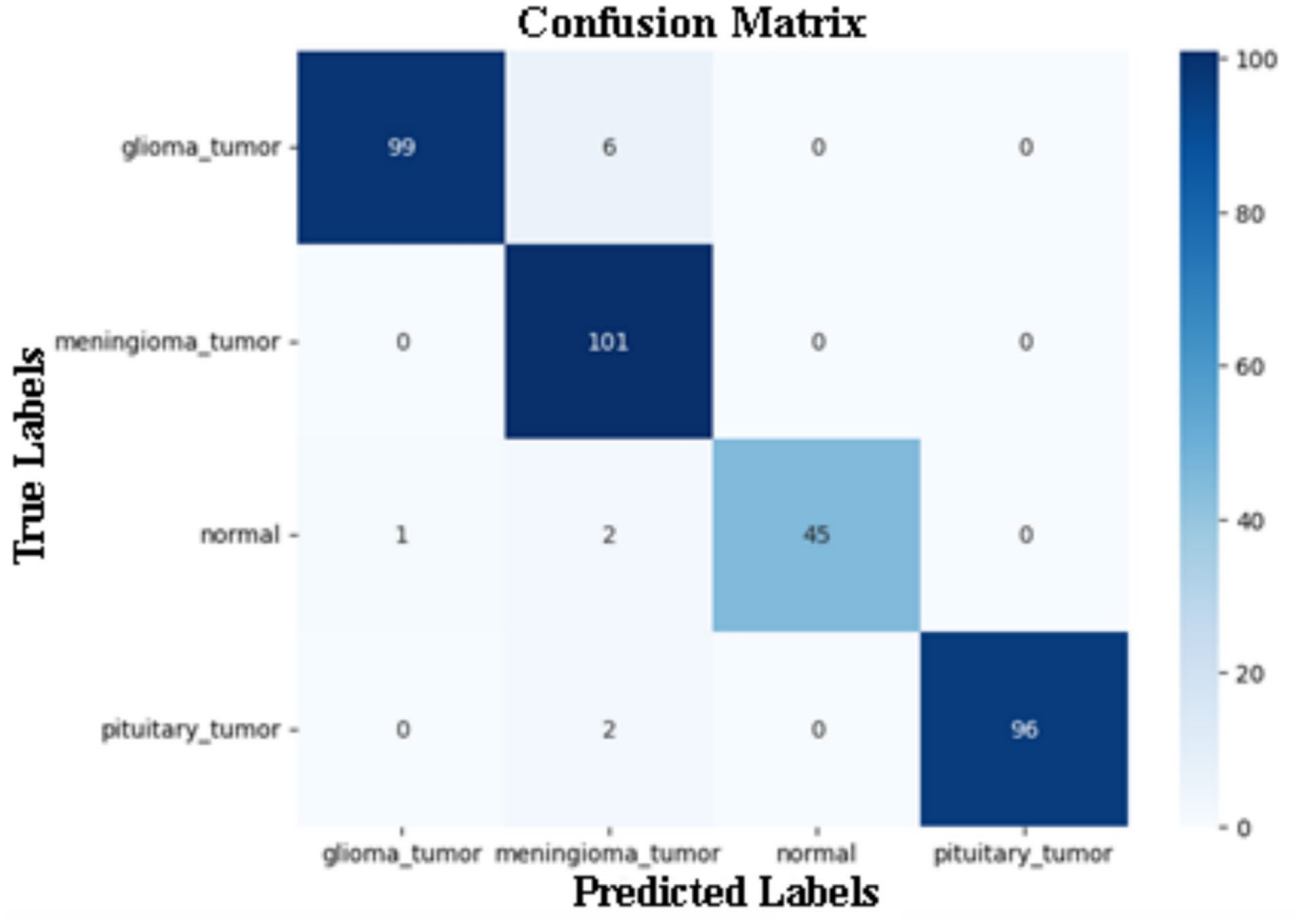
Confusion matrix for tumor classification model.

Figure 3 illustrates a representative confusion matrix obtained from one cross-validation fold. The diagonal dominance indicates correct classification across all four classes: glioma tumor, meningioma tumor, pituitary tumor, and normal brain images. The model demonstrated excellent performance in classifying glioma tumor cases, with 99 out of 105 images correctly identified. However, 6 glioma cases were misclassified as meningioma tumors. For the meningioma tumor class, the model achieved near-perfect accuracy, correctly identifying 101 out of 101 samples, with no misclassifications into other classes. In the normal category, 45 of the 48 images were correctly predicted. There were minimal errors, with one normal image classified as glioma and two as meningioma. Finally, pituitary tumors were accurately identified in 96 out of 98 cases, with only two images misclassified as meningioma tumors.

Table 4 presents a per-class classification report obtained from a representative evaluation of the proposed CNN model. The table reports precision, recall, and F1-score for each tumor category, along with the corresponding number of samples (Support). These metrics are provided to illustrate class-wise prediction behavior and should be interpreted in conjunction with the cross-validation results reported in Table 1.

**Table 4:** Classification report of prediction section.

Classes	Precision	Recall	F1-Score	Support
Glioma tumor	1.00	1.00	1.00	105
Meningioma tumor	0.91	1.00	0.95	101
Normal	1.00	0.94	0.97	48
Pituitary tumor	1.00	0.98	0.99	98
Macro Avg. accuracy	0.97	0.96	0.97	352
Weighted Avg. accuracy	0.97	0.97	0.97	352

For the glioma tumor class, high precision and recall values indicate that the model demonstrates strong discriminatory capability for this tumor type within the evaluated subset. Similarly, the meningioma tumor class shows high recall, with slightly lower precision, reflecting limited confusion with visually similar tumor categories. This observation is consistent with known radiological overlaps between glioma and meningioma appearances. The normal class achieves high precision with moderately lower recall, suggesting that while predicted normal cases are reliable, a small number of normal images are occasionally misclassified as tumorous. For pituitary tumors, the model exhibits consistently strong performance across all reported metrics, indicating effective feature extraction for this tumor type. Macro-averaged and weighted performance metrics demonstrate balanced behavior across classes, suggesting that the model does not disproportionately favor any single category despite differences in class distribution. These class-wise results complement the aggregated cross-validation analysis and provide additional insight into error patterns at the class level, rather than serving as standalone evidence of clinical performance.

### 3.3 Qualitative prediction examples

**Figure 4:**
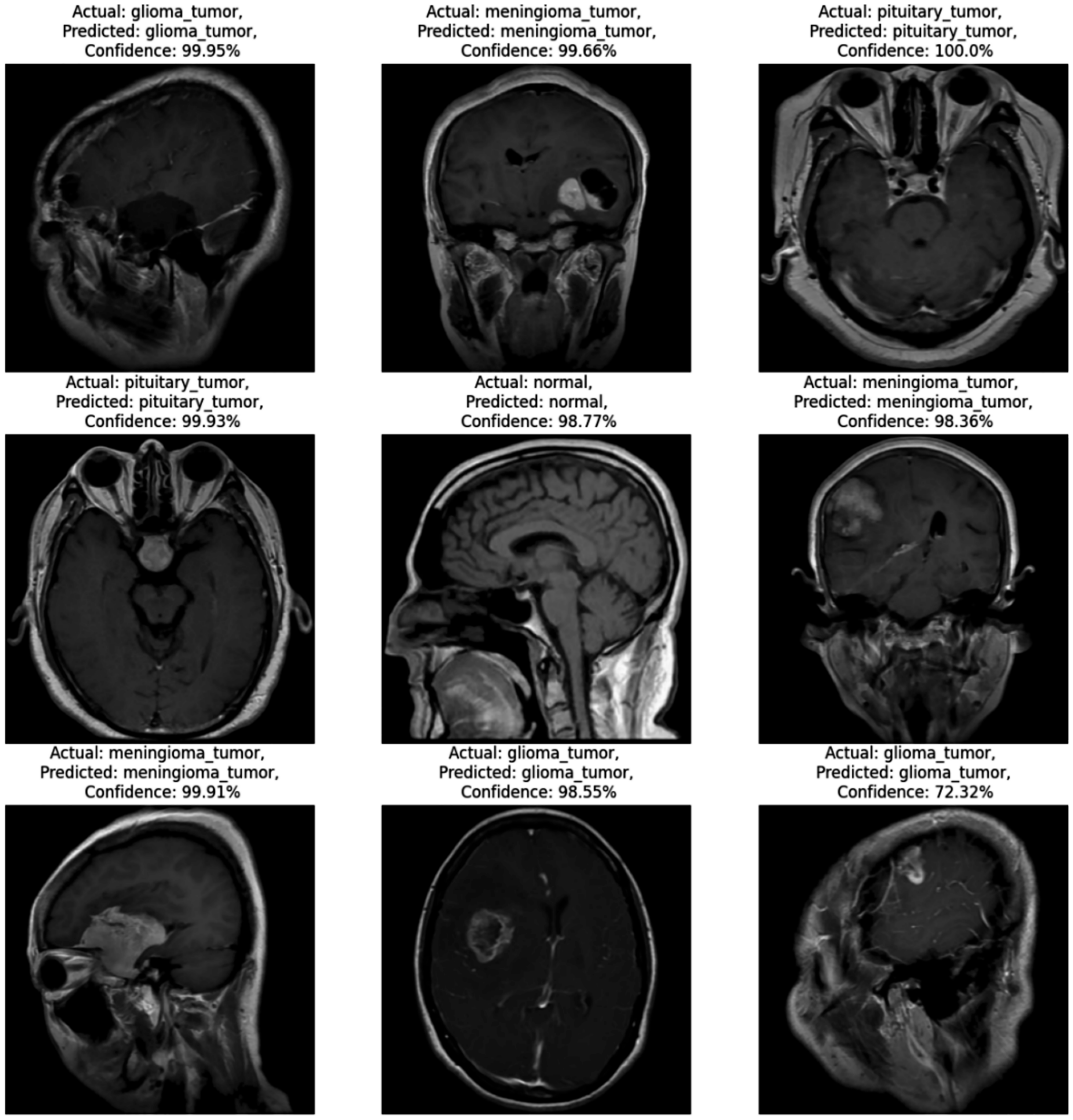
Example MRI predictions: actual vs. predicted tumor types with model confidence levels.

Figure 4 illustrates a selection of prediction results generated by the proposed CNN model, showcasing its ability to accurately classify various types of brain MRI images. Each sub-image includes the actual class label, the predicted label, and the associated prediction confidence score. The figure presents examples from all four categories: glioma tumor, meningioma tumor, pituitary tumor, and normal brain images. These qualitative examples are provided to complement the quantitative cross-validation results reported in Table 1 and to illustrate typical model outputs.

In the majority of the examples shown, the predicted labels match the ground-truth labels, with confidence levels exceeding 98% in most cases. For instance, the model correctly identifies glioma tumors with high confidence (e.g., 99.95%, 98.55%) and similarly achieves 100.0% confidence in detecting a pituitary tumor, highlighting its robustness in high-certainty predictions. Additionally, the model successfully classifies normal brain images with a confidence of 98.77%, demonstrating its ability to distinguish between healthy and pathological cases effectively. Notably, even in cases where confidence scores are relatively lower, such as the glioma tumor prediction at 72.32%, the model still maintains correct classification. This implies that the model can make accurate predictions even when image features are potentially ambiguous or less distinct. However, the displayed confidence scores are included for qualitative illustration only and should not be interpreted as calibrated clinical probabilities. Therefore, these examples should be interpreted as representative outputs rather than standalone evidence of clinical performance.

### 3.4 FastAPI-based deployment results

**Figure 5:**
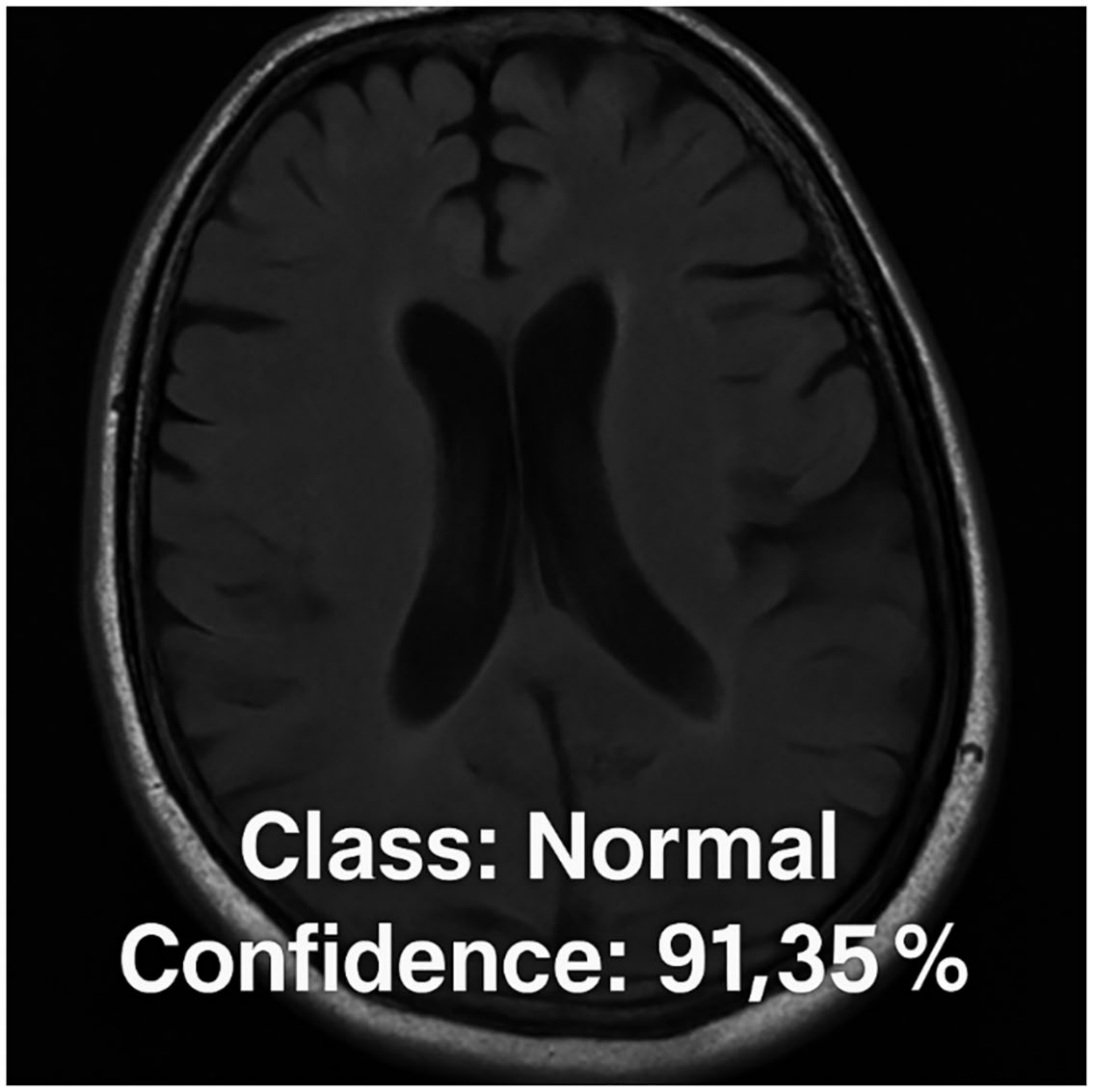
Example of the FastAPI server response: the classification output from the CNN model (“Normal” with 91.35% confidence) is overlaid on the input MRI brain scan and returned as the final response image.

Upon receiving an MRI brain image via an HTTP request, the FastAPI server initiates a processing pipeline that integrates seamlessly with a custom-trained CNN model specifically designed for brain tumor classification. The submitted image undergoes necessary preprocessing steps including resizing, normalization, and format conversion—before being fed into the CNN for inference. The model then analyzes the scan and produces a classification result along with a confidence score. In the illustrated example, the system predicts the image as “Normal” with an associated confidence score of 91.35%. To provide immediate and intuitive feedback, the classification output is programmatically overlaid on the original MRI scan using a clean and professional format. This augmented image, which includes both the predicted class and confidence score, is then returned to the user as the final API response. This example illustrates the functional integration of model inference within a web-based deployment pipeline, rather than serving as a standalone indicator of diagnostic performance (see Figure 5).

Following the classification step, the system optionally generates a Grad-CAM heatmap visualization for the same input image. The heatmap highlights spatial regions within the MRI scan that contributed most strongly to the model’s prediction, offering qualitative insight into the model’s internal feature utilization. The inclusion of Grad-CAM outputs aims to enhance transparency and interpretability of the inference process rather than to provide clinical validation of the prediction.

### 3.5 Concurrency stress test results

To evaluate the scalability characteristics of the proposed FastAPI-based deployment framework, controlled stress testing was conducted under increasing levels of concurrent requests. Each test scenario consisted of 500 total requests distributed across predefined concurrency levels. Table 5 summarizes the measured throughput, latency distribution, and failure rates for each concurrency setting.

**Table 5:** Concurrency stress test results (500 total requests per setting).

Concurrency	Throughput (req/s)	Mean Latency (ms)	p50 (ms)	p95 (ms)	p99 (ms)	Max (ms)	Failures (%)
10	12.75	773.89	750.57	908.28	1569.64	1610.60	0
50	13.10	3680.77	3631.64	4379.72	4473.73	4523.02	0
100	14.78	6015.39	2741.40	21152.87	25075.83	25705.56	8

The results indicate that system throughput remains relatively stable across concurrency levels, suggesting that inference performance is primarily limited by CPU-bound processing capacity. As concurrency increases, mean latency and high-percentile response times (p95 and p99) increase substantially, reflecting queueing effects under heavier loads.

At 100 concurrent requests, a modest failure rate (8%) was observed, indicating performance saturation under high-load conditions. While the framework demonstrates stable behavior under moderate concurrency levels, these findings suggest that large-scale clinical deployment would benefit from hardware acceleration (e.g., GPU-based inference) or containerized horizontal scaling strategies to ensure consistent latency under intensive workloads.

### 3.6 Grad-CAM Explainability results

**Figure 6:**
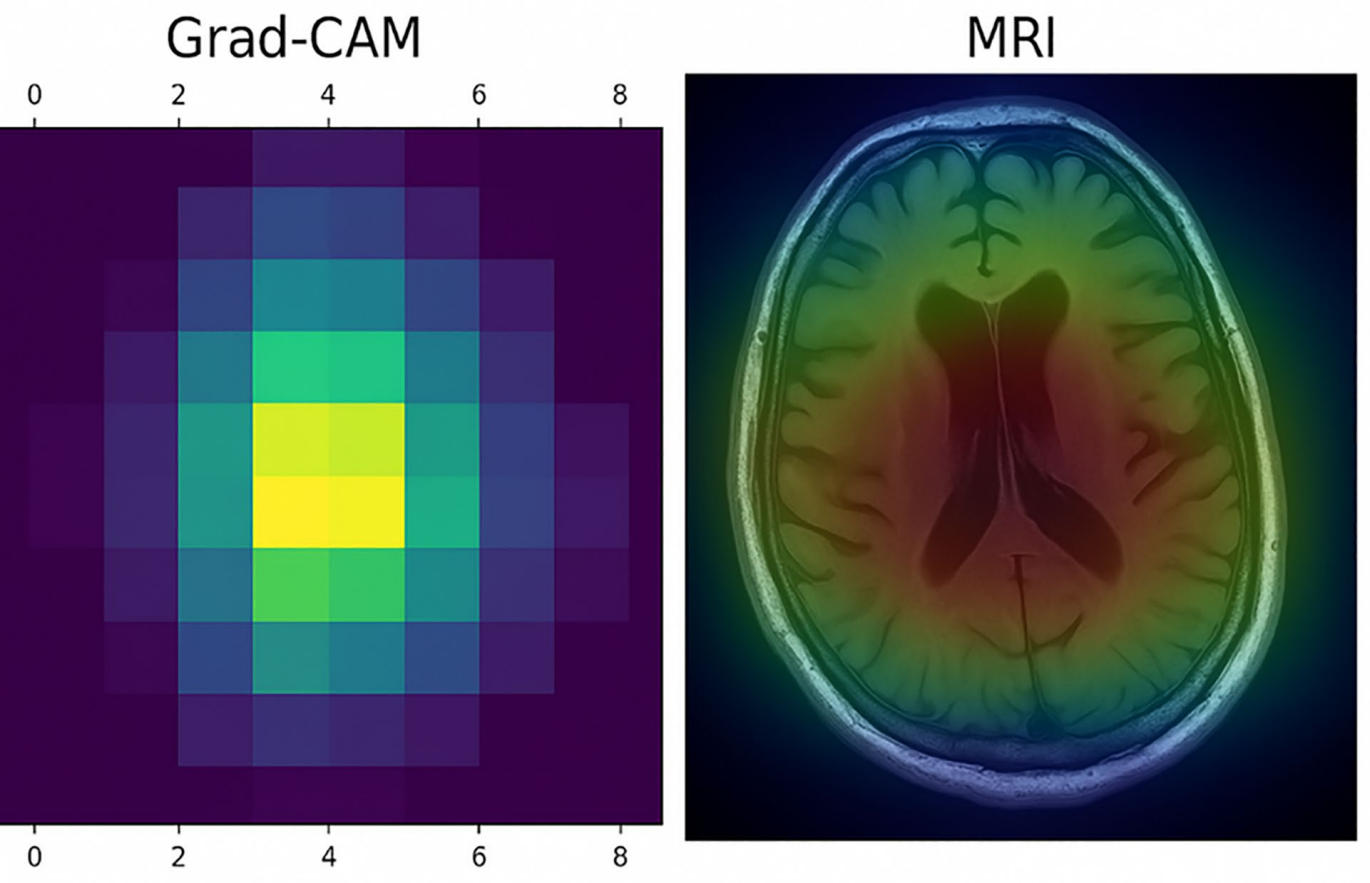
Visualization of Grad-CAM and corresponding MRI with heatmap overlay.

Figure 6 illustrates the output of the CNN model, which was deployed via a FastAPI-based server to enable real-time interpretability of medical imaging data. The left panel displays the Grad-CAM heatmap, representing the model’s attention across spatial regions of the input. Warmer colors (yellow green) correspond to areas with higher activation values, indicating greater relevance to the model’s prediction. The right panel presents the Grad-CAM heatmap overlaid on the original brain MRI, providing anatomical context to the activation patterns.

These visualizations are intended to offer qualitative insight into the model’s attention mechanisms and should be interpreted as exploratory explainability outputs. While the highlighted regions generally correspond to anatomically plausible areas, no formal clinical or expert-driven validation of the Grad-CAM results was performed in this study. Accordingly, the visualizations are presented to support transparency and understanding of model behavior rather than to assert clinical correctness. The server-side pipeline efficiently generates both the raw Grad-CAM mask and the masked MRI image on-the-fly, demonstrating the feasibility of integrating explainability mechanisms into real-time AI deployment workflows. This capability supports scalable and interpretable system design, which is a critical requirement for future clinical-facing AI applications.

## 4 Discussion

The results presented in this study demonstrate that the proposed CNN-based framework, integrated with a FastAPI deployment system, provides a technically robust and practically deployable approach for brain tumor classification using MRI images. Rather than relying on a single train–test split, model performance was assessed using stratified 5-fold cross-validation, enabling a more reliable estimation of generalization performance across different data partitions. The framework demonstrated stable and competitive performance across multiple evaluation metrics, particularly when compared with state-of-the-art transfer learning architectures.

While earlier single-split evaluations yielded near-perfect class-wise metrics, cross-validation revealed more conservative yet consistent performance estimates, underscoring the importance of robust validation strategies in medical imaging tasks. In this context, the observed classification behavior particularly the partial confusion between glioma and meningioma classes is consistent with prior findings highlighting radiological overlap among certain tumor types (Abdusalomov et al., 2023; Disci et al., 2025).

The comparative analysis indicates that transfer learning models such as ResNet50 and DenseNet121 outperform the custom CNN in terms of average classification accuracy and macro-F1 score, benefiting from pretrained feature representations learned from large-scale datasets. However, the custom CNN achieves competitive macro-AUC values despite being trained from scratch, suggesting that it effectively captures discriminative tumor-related features even under limited data conditions. From a deployment perspective, this performance–complexity trade-off is particularly relevant for real-time and resource-constrained scenarios, where inference latency and computational efficiency are critical considerations.

In contrast to prior studies that focus primarily on classification accuracy (Alam et al., 2024), the present work emphasizes end-to-end system integration, combining model inference, explainability, and deployment within a unified framework. The use of FastAPI distinguishes this study from approaches limited to offline evaluation, enabling real-time inference through a lightweight web interface. Recent studies have highlighted the suitability of FastAPI for production-grade machine learning systems due to its asynchronous request handling and efficient validation mechanisms (Narayanan, 2024). Our implementation builds upon this paradigm by incorporating on-demand Grad-CAM visualizations directly into API responses to enhance system transparency.

The deployment framework was further evaluated under concurrent request conditions to assess scalability characteristics. Stress testing demonstrated relatively stable throughput across concurrency levels, indicating CPU-bound inference behavior. As concurrent load increased, response latency rose substantially particularly at higher percentiles reflecting queueing effects under heavy workloads. At 100 concurrent requests, a modest failure rate was observed, suggesting performance saturation under high-load conditions. These findings indicate that while the framework supports moderate concurrent usage scenarios, large-scale clinical deployment would require hardware acceleration (GPU-based inference) or containerized horizontal scaling strategies.

With respect to explainability, Grad-CAM was employed as a qualitative tool to provide visual insight into the model’s attention mechanisms. While the resulting heatmaps frequently highlight anatomically plausible regions, they provide coarse localization and are not clinically validated. As a *post hoc* explanation method, Grad-CAM does not guarantee causal faithfulness and may not fully capture feature attribution reliability. Quantitative comparison with alternative explainability techniques, such as Integrated Gradients or SHAP, was beyond the scope of the present study and represents an important direction for future research.

Despite the promising results, several limitations should be acknowledged. First, the dataset size remains relatively modest (3,097 images), and patient-level identifiers were unavailable, necessitating slice-level evaluation. Although cross-validation mitigates partition bias, future studies should incorporate larger, multi-institutional datasets to enhance generalizability (Liu and Demosthenes, 2022). Moreover, the dataset originated from a single acquisition source, which minimizes inter-scanner variability under controlled experimental conditions. Real-world deployment across heterogeneous MRI systems may introduce domain shift due to differences in intensity scaling, contrast distribution, and acquisition protocols. Although inter-scanner normalization was not required in the present study, the modular FastAPI-based deployment framework allows incorporation of preprocessing standardization steps such as intensity normalization or histogram matching prior to inference. Cross-institutional and multi-scanner validation therefore remain important directions for future investigation. Finally, full interoperability with clinical standards such as DICOM and HL7, as well as production-grade orchestration using Docker and Kubernetes, represents a necessary step toward real-world healthcare integration (Gaeta et al., 2025).

A key contribution of this work lies in the explicit transition from iterative algorithmic optimization to a holistic, system-level innovation. While traditional ‘research-only’ models often prioritize peak performance on static datasets, they frequently remain isolated from clinical workflows due to high computational overhead and a lack of interpretability. By contrast, our framework adopts a “production-ready” architecture that bridges this gap. The integration of a lightweight FastAPI backend ensures low-latency, real-time inference that is essential for high-throughput radiological environments. Furthermore, by embedding on-demand Grad-CAM heatmaps directly into the API response, we shift the model from a “black-box” predictor to an explainable diagnostic aid. This synergy between deployment efficiency and visual transparency addresses the primary barriers to clinical adoption, transforming a deep learning algorithm into a functional tool designed for the practical constraints of modern neuro-oncology.

## 5 Conclusion

This study presented the development, evaluation, and deployment of a deep learning–based framework for automated brain tumor classification from MRI images. The proposed system classifies scans into four clinically relevant categories: glioma tumor, meningioma tumor, pituitary tumor, and normal, using convolutional neural networks implemented in TensorFlow and deployed via a FastAPI-based web service.

Through stratified 5-fold cross-validation and benchmarking against multiple state-of-the-art transfer learning models, the framework demonstrated stable and competitive performance across tumor classes while maintaining suitability for real-time inference. Concurrency stress testing further validated the deployment feasibility of the system under moderate multi-user conditions, clarifying performance characteristics under increasing load.

Rather than prioritizing peak accuracy alone, the study emphasizes reproducibility, deployment feasibility, scalability characteristics, and interpretability as essential components of clinical AI systems. The integration of Grad-CAM-based visual explanations provides qualitative insight into model behavior, supporting transparency in AI-assisted image analysis. While such explainability methods do not replace expert clinical judgment, they contribute to improved understanding of model predictions and highlight areas for further investigation.

By combining model development with validated deployment and concurrency evaluation, this work moves beyond theoretical experimentation toward practical implementation. Although challenges remain regarding dataset diversity, cross-institutional validation, clinical integration, and regulatory compliance, the proposed framework establishes a solid foundation for future research aimed at translating deep learning models into real-world neuro-oncological workflows.

## Data Availability

The original contributions presented in the study are included in the article/supplementary material, further inquiries can be directed to the corresponding authors.
